# Berberine Suppresses Lung Metastasis of Cancer *via* Inhibiting Endothelial Transforming Growth Factor Beta Receptor 1

**DOI:** 10.3389/fphar.2022.917827

**Published:** 2022-06-16

**Authors:** Wenjia Tian, Huifeng Hao, Ming Chu, Jingjing Gong, Wenzhe Li, Yuan Fang, Jindong Zhang, Cunzheng Zhang, Yonghui Huang, Fei Pei, Liping Duan

**Affiliations:** ^1^ Department of Gastroenterology, Peking University Third Hospital, Beijing, China; ^2^ Department of Gastroenterology, Peking University International Hospital, Beijing, China; ^3^ Key Laboratory of Carcinogenesis and Translational Research (Ministry of Education Beijing), Department of Integration of Chinese and Western Medicine, Peking University Cancer Hospital and Institute, Beijing, China; ^4^ Department of Immunology, School of Basic Medical Sciences, Peking University, NHC Key Laboratory of Medical Immunology (Peking University), Beijing, China; ^5^ Institute of Systems Biomedicine, Peking University Health Science Center, Beijing, China; ^6^ State Key Laboratory of Natural and Biomimetic Drugs, School of Pharmaceutical Sciences, Peking University Health Science Center, Beijing, China; ^7^ Department of Pathology, Peking University Third Hospital, School of Basic Medical Sciences, Peking University Health Science Center, Beijing, China

**Keywords:** berberine, trans-endothelial migration, metastasis, TGFBR1, pancreatic cancer, endothelial barrier, endothelial cell, cancer cell

## Abstract

This study investigated the effects of berberine (BBR) on pancreatic cancer (PC) lung metastasis and explored the underlying mechanisms, using a BALB/C-nu/nu nude mouse model injected with PC cells (AsPC-1). Intragastric administration of BBR dose-dependently improves survival of mice intravenously injected with AsPC-1 cells, and reduces lung metastasis. Especially, BBR significantly reduces lung infiltration of circulating tumor cells (CTCs) 24 h after AsPC-1 cells injection. *In vitro*, tumor cells (TCs) trigger endothelial barrier disruption and promote trans-endothelial migration of CFSE-labeled TCs. BBR treatment effectively ameliorates TC-induced endothelial disruption, an effect that is diminished by inhibiting transforming growth factor-β receptor 1 (TGFBR1). Blocking TGFBR1 blunts the anti-metastatic effect of BBR *in vivo*. Mechanistically, BBR binds to the intercellular portion of TGFBR1, suppresses its enzyme activities, and protects endothelial barrier disruption by TCs which express higher levels of TGF-β1. Hence, BBR might be a promising drug for reducing PC lung metastasis in clinical practice.

## Introduction

Pancreatic cancer (PC) is a highly aggressive disease with a 5-year overall survival (OS) rate of approximately 10% (Grossberg et al., 2020). In 2020, 496,000 estimated deaths worldwide were attributed to PC (Sung et al., 2021). PC’s high mortality is related to the high incidence of metastasis. In fact, approximately 50% of patients with PC present with distant metastases at the time of diagnosis (Mizrahi et al., 2020). Moreover, many patients who undergo tumor resection will develop metastases within 4 years after surgery (Grossberg et al., 2020). Hence, prevention of metastasis is a critical strategy for ameliorating PC prognosis.

Extravasation, the process of circulating tumor cells (CTCs) entering the target organs by passing through the vascular barrier, is a key step for tumor metastasis (Fidler, 2003; Jamil and Kasi, 2020). Endothelial cells (ECs), a single layer of cells lining the inside of vessels, constitute an important barrier for preventing trans-vascular migration of tumor cells (TCs) and cancer metastasis (Wettschureck et al., 2019). Recently, studies have highlighted a crucial role of TC-EC interactions in regulating cancer metastasis (Strilic et al., 2016; Keklikoglou et al., 2019; Rodrigues et al., 2019; Chang et al., 2020). TCs have been shown to destroy the EC barrier by directly adhering and disrupting ECs or by secreting a variety of biologically active substances capable of breaking down the EC barrier (Cao et al., 2013; Tichet et al., 2015; Strilic et al., 2016; Rodrigues et al., 2019; Fu et al., 2020). Interestingly, the interactions between TCs and ECs is fundamentally involved in PC metastasis (Cao et al., 2013; Huang et al., 2017), emphasizing a therapeutic potential for regulating TC-EC interactions in preventing PC metastasis. However, few studies have identified drugs capable of preventing PC metastasis by modulating the TC-EC interaction.

Berberine (BBR), an alkaloid from *Coptis chinensis*, is often employed for the clinical treatment of gastrointestinal infectious diseases. Since the end of last century, an anti-cancer potential of BBR has been discovered (Wu et al., 1998). Further studies have shown that BBR may be effective in suppressing esophageal cancer, colon adenocarcinoma, prostate cancer, and acute lymphoblastic leukemia (Iizuka et al., 2000; Zhang et al., 2010; Li et al., 2011; Ruan et al., 2017). However, the effect of BBR against PC metastasis remains unknown. Meanwhile, cardiovascular studies report that BBR protects ECs from various injuries (Wang et al., 2009; Cheng et al., 2013), suggesting that BBR may prevent TCs from passing through the endothelial barrier by protecting ECs. However, it remains unclear whether BBR inhibits trans-endothelial migration of PC cells, thus, preventing PC metastasis by improving endothelial barrier integrity.

Transforming growth factor-β1 (TGF-β1) serves as an important mediator of TC-EC interactions and is overexpressed in various types of cancer, including PC (Arjaans et al., 2012; Gore et al., 2014). TGF-β1 disrupts the endothelial barrier by stimulating endothelial contraction and/or inducing endothelial-to-mesenchymal transition (EndoMT) (Wagener et al., 2016; Wu et al., 2020). Importantly, TGF-β1 signaling is a potential target pathway of BBR as BBR may inhibit the production of TGF-β1 as well as the phosphorylation of SMAD proteins, which are downstream molecules involved in TGF-β1 signaling (Chen et al., 2019; Huang et al., 2020). Interestingly, our previous computational screening results identified TGF beta receptor 1 (TGFBR1) as a highly-scored potential target of BBR (Chu et al., 2018). However, whether, and how, BBR regulates TC-EC interactions *via* modulating the TGF-β1 signaling pathway remains unclear.

Hence, in the current study we examined the effects of BBR on lung metastasis of PC, and explored the underlying mechanisms by focusing on the effects of BBR on protecting endothelial disruption through interacting with endothelial TGFBR1.

## Results

### Berberine Inhibits Pancreatic Cancer Lung Metastasis

**FIGURE 1:**
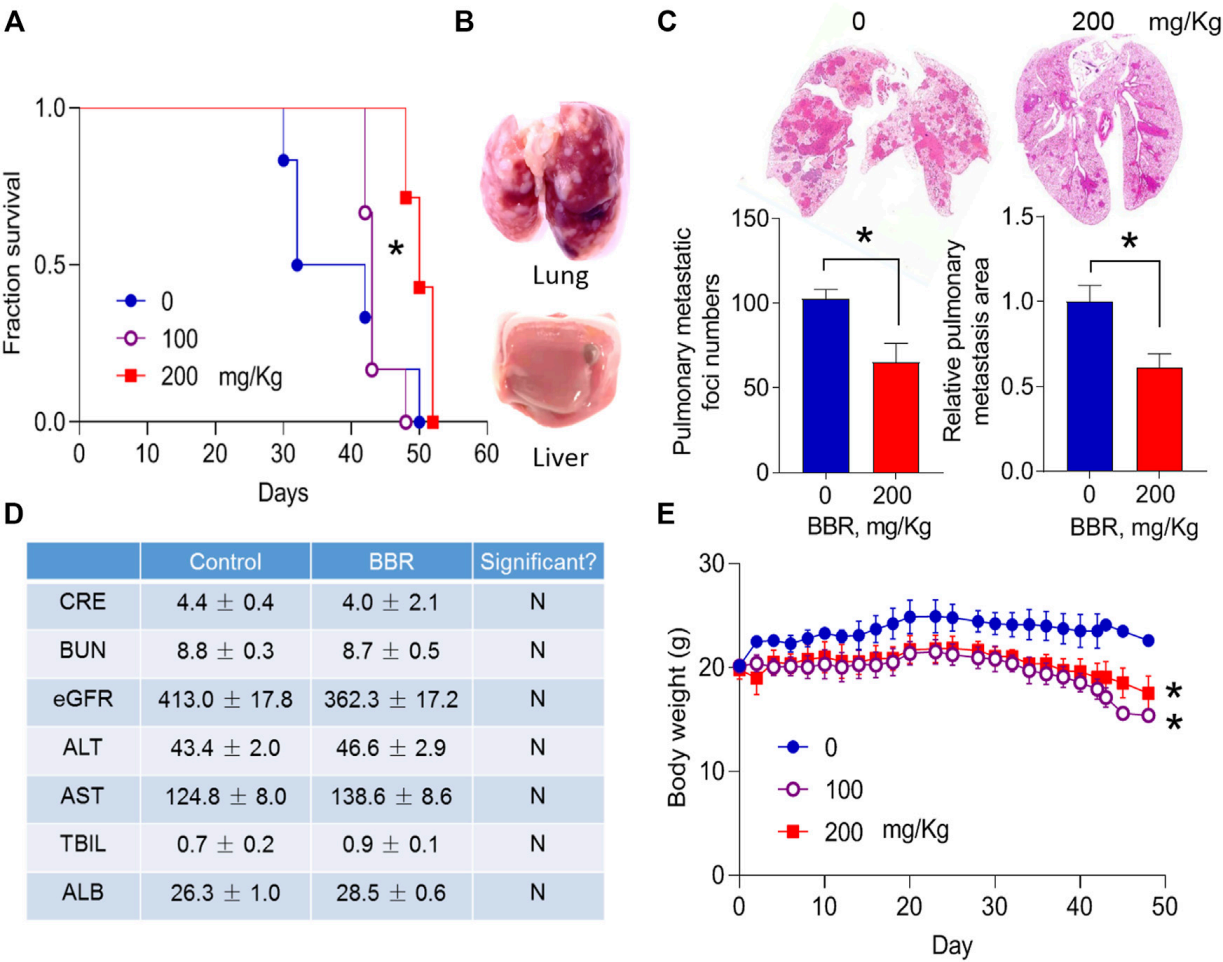
BBR inhibits lung metastasis of pancreatic cancer cells **(A)**. Effect of BBR on the survival of mice that were intravenously injected with PC cells **(B)**. Morphological changes of the resected lungs and livers from dead mice **(C)**. Effects of BBR (BBR, 200 mg/kg, oral gavage daily) on the number and total area of lung metastatic foci **(D)**. Effect of BBR on the biochemical metabolism of mice **(E)**. Body weight changes of mice during drug treatment. *p < 0.05; *n* = 6 in **(A)**, Log-rank (Mantel-Cox) test; *n* = 6 in **(C)**, *n* = 5 in **(D)**, Student’s *t-*test; *n* = 7 in **(E)**, two-way analysis of variance (ANOVA) with Dunnett’s multiple comparison test.

Hematogenous metastasis plays a significant role in PC dissemination (Franses et al., 2020). To explore the effects of BBR on PC hematogenous metastasis, BBR was intragastrically administrated to male BALB/C-nu/nu nude mice that had been injected intravenously with AsPC-1 cells through the tail vein (Tiwari et al., 2020). The OS of mice was observed. BBR (100, 200 mg/kg) treatment increased the OS of mice in a dose-dependent manner (Figure 1A). While, autopsy results confirmed severe cancer metastasis in the lungs, but not in the liver (Figure 1B).

We then determined the effects of BBR on lung metastasis of PC cells in mice that were sacrificed and examined 6 weeks after PC cells injection. BBR treatment (200 mg/kg) significantly reduced the number, and total area of metastatic nodes in the lungs by 36.5 and 35.0%, respectively (Figure 1C). No differences were identified in the plasma biochemical products of control and BBR treatment groups (Figure 1D). No overt liver or kidney injury was observed in the hematoxylin and eosin (H&E)-stained sections (Supplementary Figure S1). Body weights were slightly decreased in BBR-treated mice (Figure 1E), consistent with the previously reported weight loss effect of BBR (Lee et al., 2006).

### Berberine Inhibits Lung Infiltration of Pancreatic Cancer Cells

**FIGURE 2:**
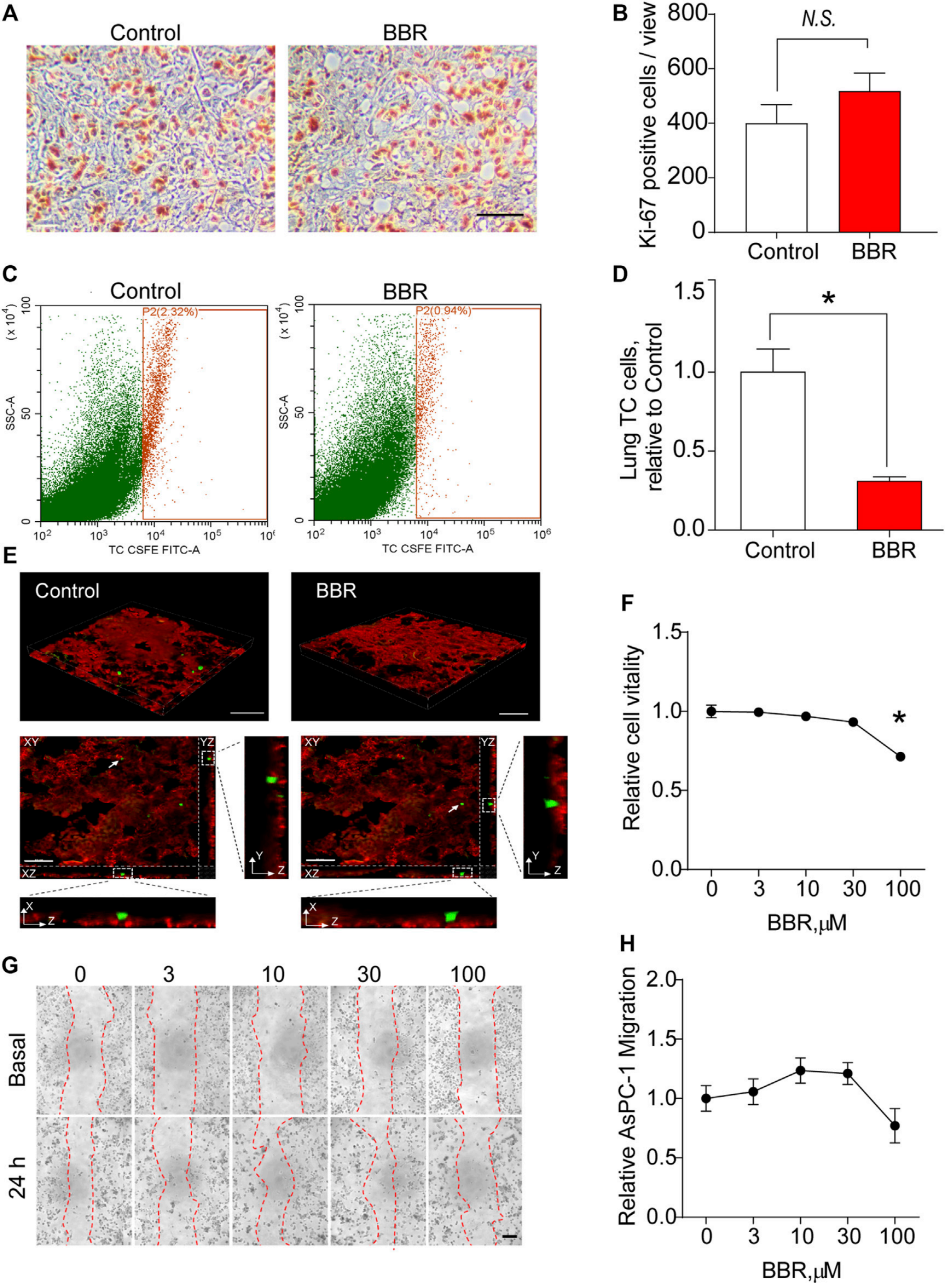
BBR inhibits lung infiltration of PC cells (AsPC-1). **(A,B)**. Representative images **(A)** and statistical results **(B)** of the Ki-67 staining of lung metastatic foci. Scale bar = 100 μm. **(C,D)** Representative images **(C)** and statistical results **(D)** of the flow cytometric analysis of lung infiltration of circulating cancer cells 24 h after the intravenous injection of CFSE-labeled AsPC-1 cells in the control or BBR-pretreated groups. **(E)** 3D reconstruction fluorescence images of mice lung tissue section dissected 24 h post-intravenous injection of CFSE-labeled AsPC-1 cells in control or BBR-pretreated groups. Lower panels showed XZ and YZ views of two tumor cells indicated by white arrows. Red: vasculature, green: CFSE-labeled AsPC-1 cells. Scale bar = 100 μm in upper panels, and 50 μm in lower panels. **(F)** Effect of BBR treatment for 24 h on the proliferation of AcPC-1 cells *in vitro*. **(G,H)**. Effect of treating AcPC-1 cells with BBR for 24 h, on the migration **(G)**; representative images; **(H)**, statistical results) of AsPC-1 cells. Scale bar = 20 μm *p < 0.05; *n* = 4 in **(B,D)**, *t*-test; *n* = 6 in **(F,H)**, one-way ANOVA with Dunnett’s multiple comparisons test.

To understand the mechanism by which BBR inhibited lung metastasis of PC cells, the metastatic foci of the two groups were stained with Ki-67, a marker of proliferating cells. No significant differences were observed in Ki-67 staining, suggesting that BBR did not exert a significant effect on PC cells proliferation in the lungs (Figure 2A,B).

Then, we examined whether BBR inhibited lung infiltration of PC cells. CFSE-labeled PC cells were intravenously injected into mice pre-treated with control or BBR for 3 days 24 h later, the mice were perfused with PBS to remove the circulating PC cells, and the lung infiltration of PC cells was quantified using flow cytometric analysis. Impressively, the results showed that BBR treatment significantly decreased PC cells accumulation in lungs (Figure 2C,D). Noticeably, 3D reconstruction fluorescence images of mice lung tissue section confirmed that most remaining PC cells in lungs were located outside of the microvessels, indicating that BBR may work by preventing PC cells from transporting from the circulation to the lung interstitial tissue (Figure 2E). To determine whether the reduction of PC accumulation in lung was resulted from a direct inhibitory effect of BBR on PC cells when PC cells were exposing to plasma BBR for 24 h, we examined the direct effects of BBR on PC cells proliferation and migration *in vitro*. The results demonstrated that PC cells proliferation and migration were not significantly influenced by BBR treatment for 24 h at regular concentrations (0–30 μM; Figure 2F–H and Supplementary Figure S2). At a concentration of 100 μM, BBR only inhibited proliferations of AsPC-1 and SW1990 cells by ∼29 and ∼21%, respectively (Figure 2F and Supplementary Figure S2). Together, these results highlighted an important role of BBR in preventing PC cells infiltration into the lungs through improving the lung vascular barrier.

### Berberine Prevents Trans-Endothelial Migration of Pancreatic Cancer Cells in an Endothelial-Dependent Manner

**FIGURE 3:**
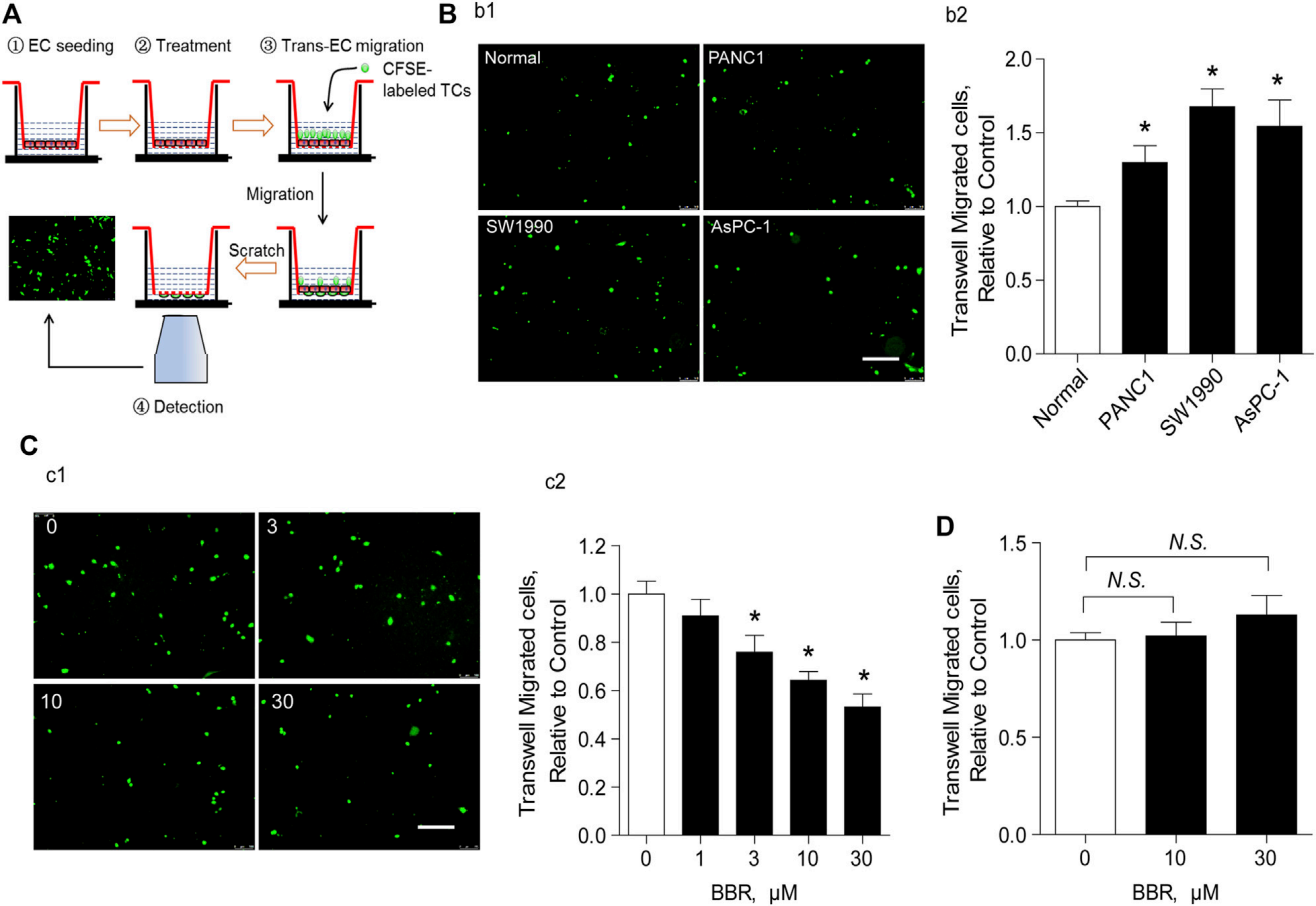
BBR prevents trans-endothelial migration of PC cells in an endothelial-dependent manner **(A)**. Schematic illustration of trans-endothelial migration of cancer cells analysis **(B)**. Representative images **(b1)** and statistical results **(b2)** of the effect of the EC layer incubation with normal or PC cells (PANC1, SW1990, and AsPC-1) on the trans-endothelial migration of CFSE-labeled AsPC-1 cells. Scale bar = 200 μm **(C)**. Representative images **(c1)** and statistical results **(c2)** of the effect of BBR treatment on the trans-endothelial migration of CFSE-labeled AsPC-1 cells. Scale bar = 200 μm **(D)**. Statistical results of the effect of BBR treatment on the trans-endothelial migration of CFSE-labeled AsPC-1 cells when ECs were absent. *p < 0.05; *n* = 5–10, one-way ANOVA with Dunnett’s multiple comparisons test.

ECs are a critical component of the vascular barrier, particularly in lung microvessels (Wettschureck et al., 2019). To explore the potential role of BBR on the vascular barrier, we investigated its effect on the trans-endothelial migration of PC cells *in vitro*. As presented in Figure 3A, after stabilizing the endothelial layer in the upper chamber, it was stimulated with CFSE-unlabeled PC cells in the presence of a vehicle or BBR. Endothelial barrier dysfunction was then assessed using the CFSE-labeled TCs. Results showed that treating the endothelial layer with PC cells (PANC1, SW1990, or AsPC-1) enhanced the trans-endothelial migration of CFSE-labeled TCs, compared to treatment with normal pancreatic cells (Figure 3B). This was indicative of increased endothelial barrier injury.

We then explored whether BBR inhibited trans-endothelial migration of AsPC-1 cells. Pre-incubation of ECs and the unlabeled cancer cells with BBR for 20 h (0–30 μM) reduced trans-endothelial migration of CFSE-labeled AsPC-1 cells in a dose-dependent manner (Figure 3C). Here, the CFSE-unlabeled cancer cells were used as a pathological stimuli to damage the EC barrier, in order to explore a direct effect of BBR on protecting EC barrier. Importantly, when ECs were absent, BBR failed to ameliorate the migration of CFSE-labeled AsPC-1 cells (Figure 3D), suggesting that ECs play a crucial role in the inhibition of AsPC-1 cells trans-endothelial migration by BBR. Notably, 20 h of incubation with BBR did not impact EC proliferation (Supplementary Figure S3). Furthermore, although EC necroptosis reportedly plays a pivotal role in promoting TC metastasis (Strilic et al., 2016), our results demonstrated that BBR did not repress EC necroptosis (Supplementary Figure S4).

### Berberine Inhibits Trans-Endothelial Migration of Cancer Cells *via* Direct Inhibition of Transforming Growth Factor-β Receptor 1

**FIGURE 4:**
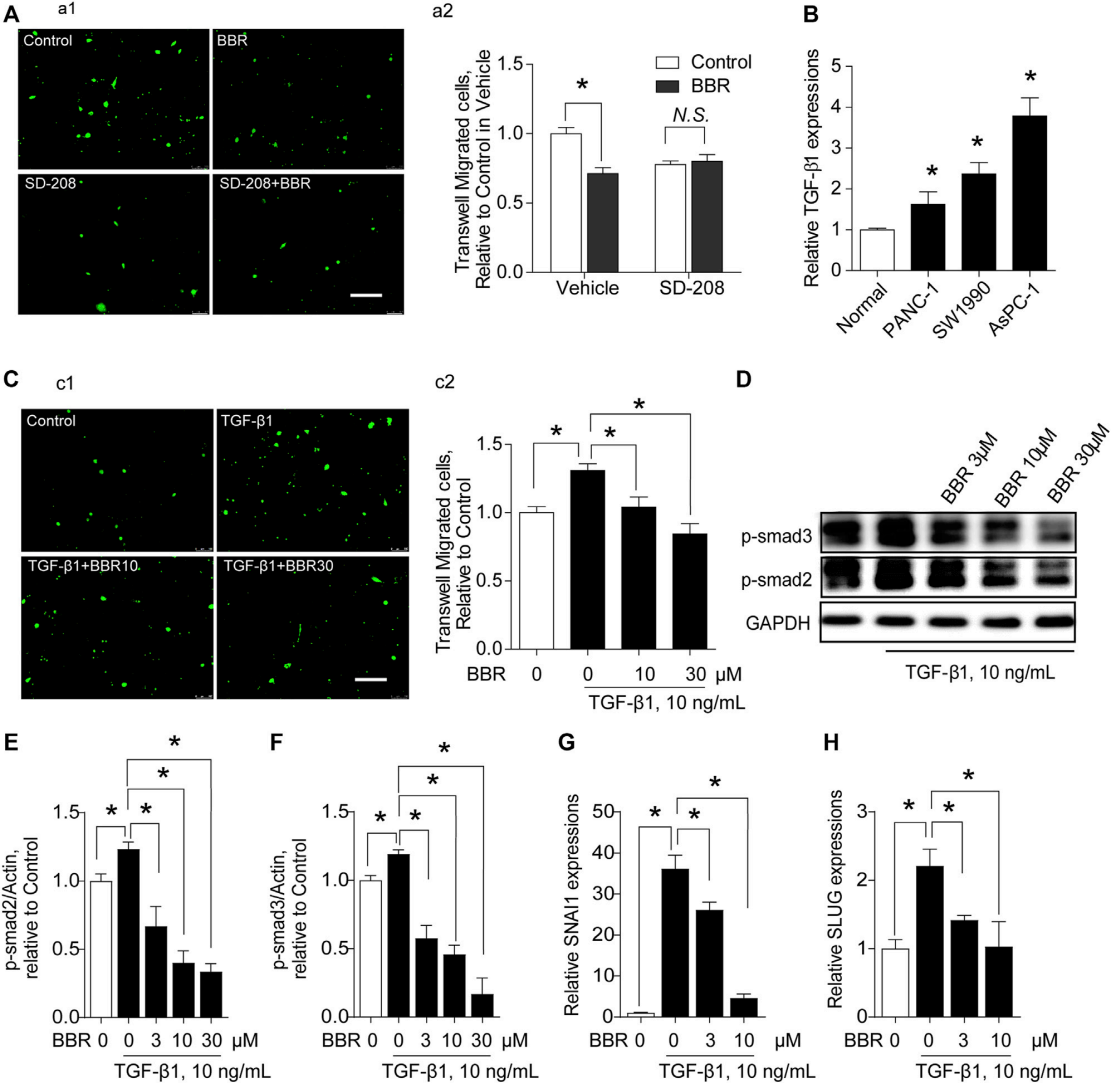
BBR prevents trans-endothelial migration of pancreatic cancer cells in a TGFBR1-dependent manner. **(A)** Effect of TGFBR1 inhibition on the modulation of BBR in the trans-endothelial migration of AsPC-1 cells. **(a1,a2)** representative images and statistical results, respectively. *n* = 10–15. **(B)**. Production of TGF-β1 in PC cells (PANC-1, SW1990, AsPC-1). **(C)**. Effect of BBR on TGF-β1-triggered trans-endothelial migration of AsPC-1 cells. **(c1,c2)** representative images and statistical results, respectively. *n* = 10–15. **(D–F)**. Representative western blot images **(D)** and statistical results **(E,F)** of the effect of BBR on TGF-β1-induced phosphorylation of SMAD2/3 in ECs. *n* = 3 **(G)**. Effect of BBR on TGF-β1-stimulated expression level of SNAI1 in ECs. *n* = 3 **(H)**. Effect of BBR on TGF-β1-stimulated expression of SLUG in ECs. *n* = 3. *p < 0.05; one-way ANOVA with Bonferroni’s multiple comparison tests.

TGFBR1 was predicted to be a highly-scored target of BBR, based on motifs-based screening in pharmacophore databases (Chu et al., 2018). Importantly, TGF-β1 has been shown to be active in inducing a disruption of endothelial barrier (Wagener et al., 2016). Hence, we explored the involvement of TGFBR1 in BBR’s suppressive effect on the trans-endothelial migration of PC cells. When TGFBR1 was inhibited, the inhibitory effect of BBR on trans-endothelial migration of AsPC-1 cells was blunted (Figure 4A), indicating that BBR prevented the trans-endothelial migration of cancer cells by interrupting TGF-β1 signaling. Indeed, PC cells expressed significantly higher levels of TGF-β1 compared with normal pancreatic ductal epithelia (Figure 4B). And, incubating ECs with TGF-β1 increased the trans-endothelial migration of AsPC-1 cells, an effect that was inhibited by BBR (Figure 4C). Moreover, SMAD2/3, SNAI1, and SLUG were all classical substream molecules of TGF-β1 signaling. BBR treatment dose-dependently inhibited the phosphorylations of SMAD2/3 and the upregulations of SNAI1 and SLUG (Figures 4D–H), further emphasizing the inhibitory effect of BBR on TGFBR1.

**FIGURE 5:**
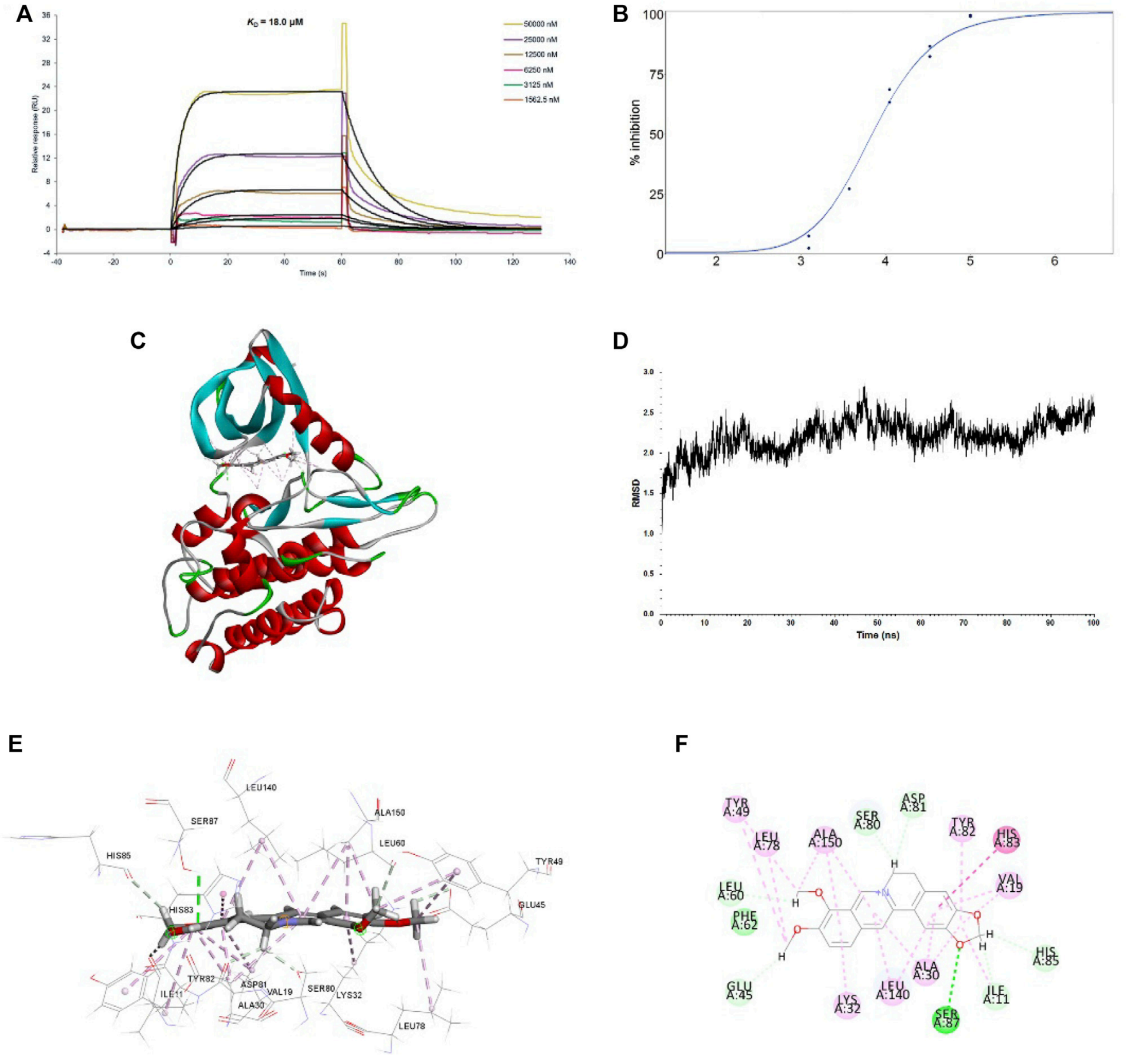
Interactions between BBR and TGFBR1 **(A)**. Binding affinity of BBR to TGFBR1. The binding curves were analyzed using a kinetic analysis supplied with Biacore Evaluation Software. Data are presented as RU over time (s). The *K*_D_ were calculated using a 1:1 binding model **(B)**. Inhibition of BBR on TGFBR1 kinase activity. The dose-response curve for IC_50_ value was determined by nonlinear regression **(C)**. Binding feature of BBR with TGFBR1. BBR and key residues are shown as sticks with carbon (gray), oxygen (red), and nitrogen (blue). Electrostatic interaction is shown as dashed lines with π-π (purple), π-alkyl (pink), and hydrogen bonds (green). Secondary structural elements are depicted as ribbons (coils, α-helices; arrows, β-sheets). Color is based on secondary structures (α-helices, red; β-sheets, blue; loops, green) **(D)**. molecular dynamics (MD) simulation of BBR in complex with TGFBR1. The selected pose was subjected to 100 ns MD simulations using a standardized MD protocol through PP. Stability was analyzed by root mean square deviation (RMSD) vs. time (ns) **(E)**. Molecular interaction of BBR with TGFBR1 residues (shown in sticks and labeled in black) **(F)**. Molecular interaction schemes of BBR with TGFBR1 residues.

We then examined whether there was a direct interaction between BBR and TGFBR1 using surface plasmon resonance (SPR) and molecular docking strategies. Our results showed that BBR bound to TGFBR1 with an equilibrium dissociation constant (*K*_D_) of 18.0 μM (Figure 5A). Moreover, the TGFBR1 kinase activity assays revealed that BBR inhibited the kinase activity of TGFBR1 in a dose-dependent manner with an IC_50_ of 7.056 μM (Figure 5B). Furthermore, the molecular docking results demonstrated that BBR was docked into the active site of TGFBR1 in a stable state (Figure 5C,B). Moreover, molecular modeling results revealed that BBR interacted with the key residues in the active site of TGFBR1, including Glu45, Tyr49, Asp81, Tyr82, and His83 (Figure 5E,F).

### Transforming Growth Factor-β Receptor 1 is a Functional Target of Berberine in the Reduction of Pancreatic Cancer Cells Lung Metastasis *in vivo*

**FIGURE 6:**
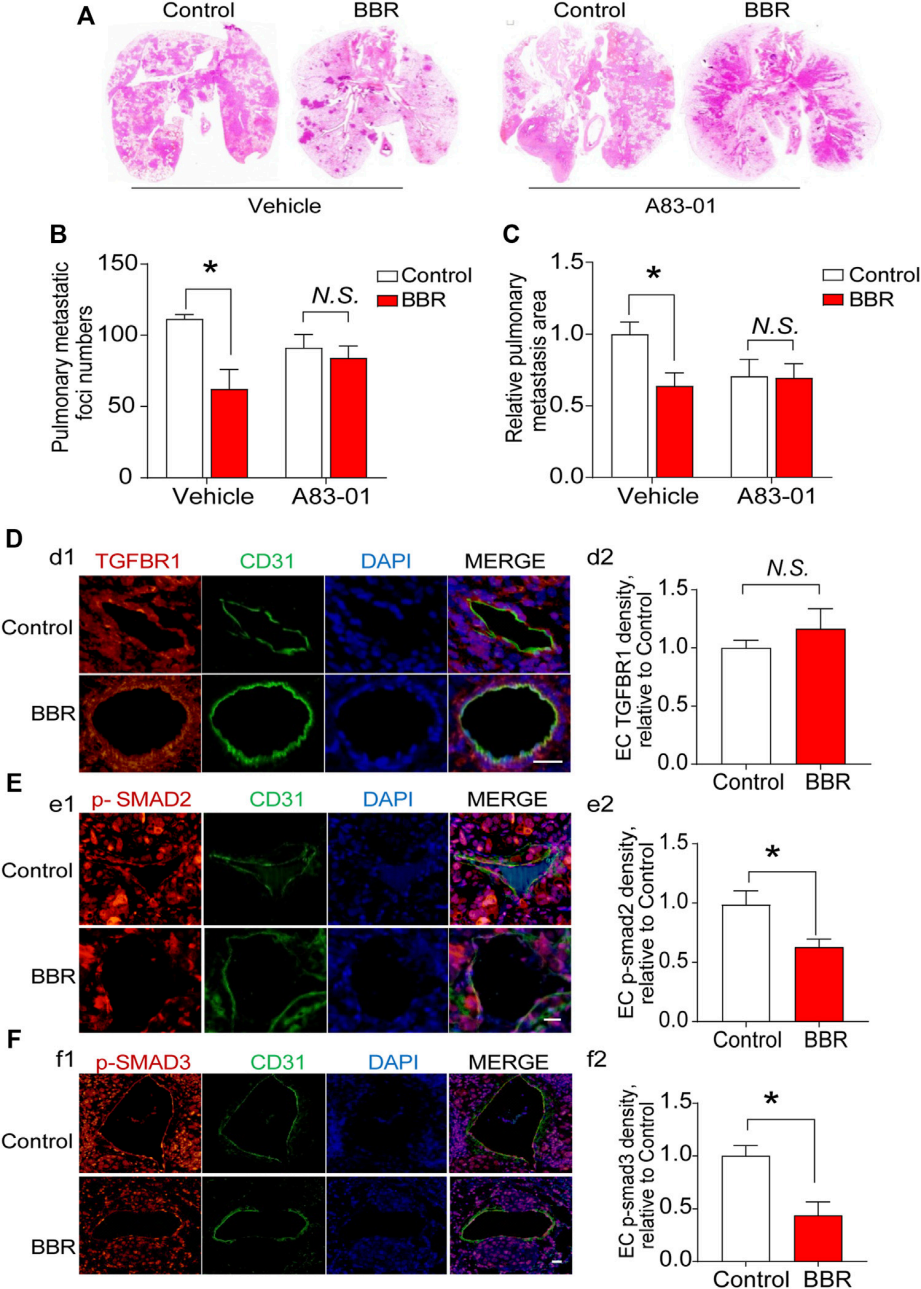
TGFBR1 mediates the anti-metastatic effect of BBR *in vivo* **(A)**. Representative images, and **(B)**. statistical results of the number and **(C)**. area of lung metastatic foci. **(D–F)** Immunofluorescent staining and endothelial cells (CD31; green) was performed in pulmonary vessels of mice in control and BBR groups. DAPI stains nuclei in blue. Representative immunofluorescent staining images and statistical results of the effects of BBR on endothelial TGFBR1 density **(d1–d2; red)**, SMAD 2/3 phosphorylation **(e1–e2, f1–f2; red)**. Scale bar = 25 μm *p < 0.05; Student’s *t*-test; *n* = 5 in **(D–F)**.

We then determined whether TGFBR1 participated in the anti-metastatic effect of BBR *in vivo*. A83-01 (10 mg/kg/day) was used to inhibit the activation of TGFBR1 *in vivo* (Chen et al., 2015). BBR suppressed lung metastasis of PC cells in mice treated with the A83-01 vehicle, whereas the anti-metastatic effect of BBR was significantly reduced in mice receiving A83-01 (Figures 6A–C). Notably, no differences were observed in serum TGF-β1 levels in the vehicle- or A83-01-treated groups (Supplementary Figure S5), and BBR did not influence the expressions of TGFBR1 in ECs which were identified by CD31-positive staining (Figure 6D). However, BBR treatment significantly suppressed SMAD 2/3 phosphorylation in ECs but not in the background tumor cells (Figure 6E,F and Supplementary Figure S6), indicating that BBR inhibited TGF-β1 signaling *in vivo*.

## Discussion

**FIGURE 7:**
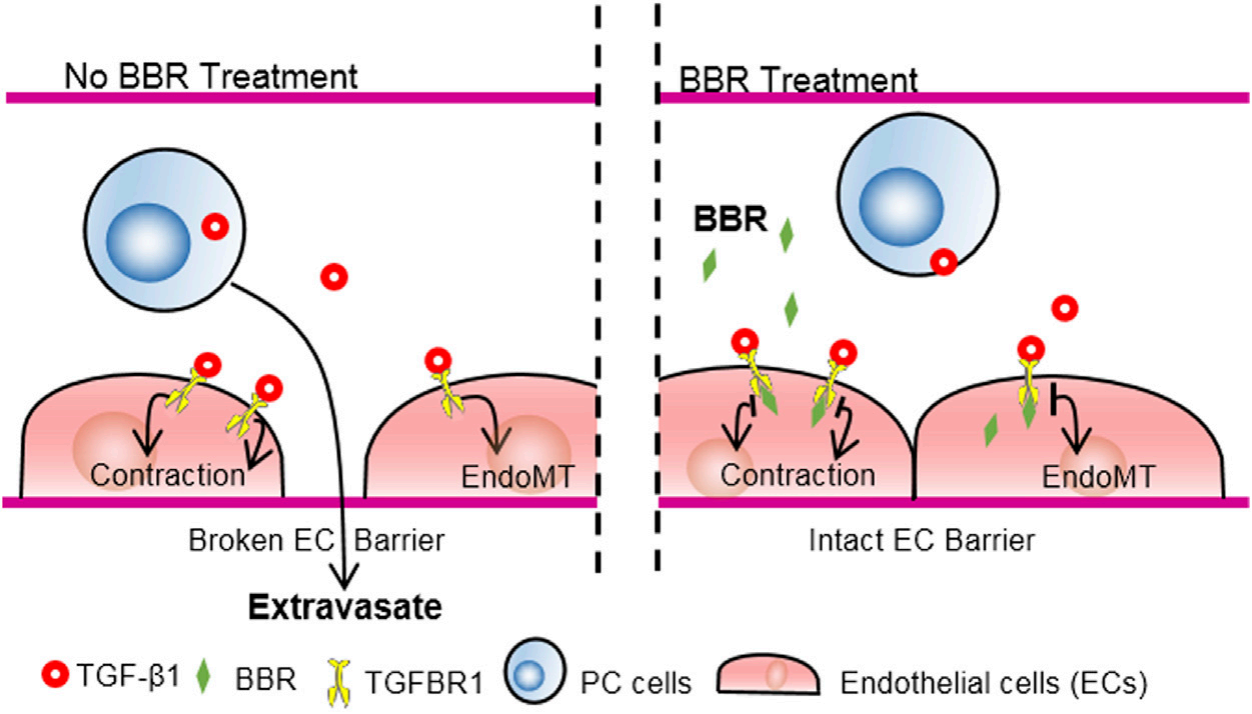
Schematic illustration of the anti-metastatic effect of BBR *via* TGFBR1 inhibition. PC cell-derived TGF-β1 disrupts the EC barrier through stimulating TGFBR1, a receptor for TGF-β1, and promotes PC metastasis. BBR interacts with the intracellular portion of TGFBR1, inhibits the enzymatic activities of TGFBR1, improves EC barrier, and suppresses PC metastasis.

In the present study, we determined the role of BBR in preventing lung metastasis of PC. We found that administration of BBR, at clinically relevant concentrations, effectively reduced both the number and total area of metastatic foci in the lungs and improved survival of mice. BBR treatment did not significantly affect PC cells proliferation, however, it suppressed lung infiltration of circulating PC cells. *In vitro*, BBR inhibited trans-endothelial migration of PC cells in an endothelial-dependent manner, which was blunted by inhibiting TGFBR1, the type 1 receptor of TGF-β1. Further, it was revealed that BBR directly bound TGFBR1 and suppressed TGF-β1 signaling. Together, the results demonstrated that BBR may suppress lung metastasis of PC by ameliorating TGF-β1-mediated endothelial barrier disruption through binding to endothelial TGFBR1 and repressing endothelial TGF-β1 signaling, as schematically summarized in Figure 7.

BBR has become popular due to its therapeutic effects in various conditions, such as intestinal inflammation, hypertension, hyperlipidemia, diabetes, and cancer (Kong et al., 2004; Li et al., 2020; Qin et al., 2020; Samadi et al., 2020; Cai et al., 2021). PC is a highly aggressive cancer with a high incidence of metastasis (Mizrahi et al., 2020). However, it is unknown whether BBR can restrain PC metastasis. Since hematogenous metastasis was fundamentally involved in PC dissemination (Franses et al., 2020), we determined the effects of BBR on PC metastasis using a classical mouse model of TC hematogenous metastasis (Tiwari et al., 2020). Impressively, BBR treatment not only inhibited lung metastasis of PC cells, but also improved the mouse survival, highlighting a protective role of BBR in PC. Notably, BBR was administered at clinically relevant concentrations, and no toxic effects were observed in BBR-treated mice. Thus, our results highlighted that BBR may inhibit PC metastasis effectively and safely, and will be valuable for supporting the clinical application of BBR to combat PC metastasis.

Previous anti-cancer studies on BBR mainly focused on the inhibitory effect of BBR on cancer cells (Samadi et al., 2020). Interestingly, in the present study, ECs were significantly involved in BBR preventing PC metastasis. First, although BBR significantly inhibited lung metastasis of PC, the proliferative status of PC cells in lung metastatic foci was not altered by BBR. Second, the lung infiltration of circulating PC cells was markedly suppressed in BBR-pretreated mice. However, when BBR was administered *in vitro* at regular concentrations (0–30 μM), it had no direct influence on proliferation or migration of PC cells, suggesting that the anti-infiltration effect of BBR was PC-cell-independent. Third, BBR had an inhibitory role on the trans-endothelial migration of PC cells *in vitro* analysis. However, this effect was not observed when ECs were removed from the system. Together, these results emphasized that ECs played a critical role in mediating the anti-metastatic actions of BBR, which is in line with recent findings that modifying EC barrier is sufficient to modulate cancer metastasis (Strilic et al., 2016).

Metastasis poses a significant challenge to clinical cancer therapy. Although the TC-EC interaction is reportedly crucial for mediating cancer metastasis (Tichet et al., 2015; Strilic et al., 2016; Huang et al., 2017; Wettschureck et al., 2019; Fu et al., 2020), few clinical drugs capable of effectively suppressing cancer metastasis by regulating TC-EC interactions are available. Considering that BBR restricted PC lung metastasis through endothelial protection, it may also prove effective for treatment of other cancer cells, particularly those expressing high levels of TGF-β1. Thus, this study may promote BBR as a candidate for combating metastasis of several kinds of cancers by modifying TC-EC interactions.

Previous studies have demonstrated that TGF-β1 signaling promotes cancer metastasis *via* disrupting the endothelial barrier (Fu et al., 2020). BBR has also been shown to be effective in inhibiting TGF-β1 signaling (Chen et al., 2019; Huang et al., 2020), however, the detailed underlying mechanisms remain obscure. Here, we identified TGFBR1 as an important target of BBR. When TGFBR1 was inhibited, the effects of BBR on trans-endothelial migration of PC cells *in vitro* and PC lung metastasis *in vivo* were both significantly reduced, suggesting a critical involvement of TGFBR1 in BBR inhibiting trans-endothelial migration and lung metastasis of PC cells. These observations were further supported by the evidences that BBR directly interacted with TGFBR1 at its key residues in the active site and effectively reduced its enzyme activity. Together, the present study confirms that TGFBR1 is a novel target of BBR, which extends our understanding of the pharmacological actions of BBR in suppressing lung metastasis of cancer cells.

This study has some limitations. First, in this cancer metastasis model, we observed overt lung metastases rather than liver metastases for PC due to the injection of PC cells *via* tail vein, instead of portal vein. In such cases, PC cells directly entered into the blood stream (Fidler, 2003), and eventually, the cancer cells preferably seed on lung. Since lung is also an important target organ of PC metastasis in clinical conditions (Yachida et al., 2010), the present study can only confirm an effect of BBR on retarding PC metastasis to lung. However, it may provide implications on the effects of BBR in preventing lung metastasis of other kinds of cancer cells. Second, gut microbiome is an important target of BBR. Recently, we have demonstrated that BBR ameliorates ovariectomy-induced anxiety-like behaviors and visceral hypersensitivity by regulating the gut microbiome (Fang et al., 2021; Zhang et al., 2021). In the current study, we cannot fully exclude the possibility that the gut microbiome might be involved in BBR preventing PC lung metastasis However, we propose that the direct modulation of BBR on TC-EC interaction might play a major role in the anti-metastatic effect of BBR. For one thing, solid evidences showed that BBR directly bound to TGFBR1, inhibit TGF-β1 signaling, and reduced trans-EC migration of PC cells. For another, the direct effects of BBR on ECs have already been proven to be a reliable mechanism for improving vascular system *in vivo* (Cai et al., 2021). Third, the present study is limited to explore the effects and mechanisms of BBR on retarding PC lung metastasis using a classical hematogenous metastasis model which is less related to primary PC cells growth in pancreases. Thus, effects of BBR on regulating primary PC cells growh in pancreases were not investigated here. These limitations may warrant further studies in the future.

In summary, the present study revealed the effects and detailed mechanisms of BBR in preventing PC lung metastasis. BBR directly binds TGFBR1, suppresses TGF-β1 signaling, inhibits TC-EC interactions, and retards lung metastasis of PC in a novel endothelial-related manner. This study is valuable as it deepens our knowledge on the anti-cancer roles of BBR, and is theoretically instrumental for clinical PC therapy. Further in-depth investigations and clinical trials are required to verify the anti-metastatic effects of BBR in cancer patients.

## Methods

### Reagents

BBR, with a purity of over 98%, was purchased from Beijing Beiluo Biotechnology Co., Ltd. (Beijing, China). BBR was dissolved in dimethyl sulfoxide (DMSO) at a concentration of 100 mM. The solution was further diluted in distilled water to a concentration of 10 mM of BBR. Thoroughly dissolved BBR solution at concentrations of 10 and 30 μM were finally prepared in culture medium immediately before being added to cells. To treat mice *via* oral gavage, BBR was dissolved in distilled water (Fang et al., 2021). SD-208 and A83-01 were purchased from Selleck Company (TX, United States). Human recombinant TGF-β1 was obtained from Sino Biological Company (Beijing, China).

### Mice

Male immunodeficient BALB/C-nu/nu nude mice aged 6–8 weeks were purchased from Beijing Vital River Laboratory Animal Technology Co., Ltd. (Beijing, China) and were housed in a specific pathogen free (SPF) environment with free access to food and water. All mice were subjected to an acclimatization period of at least 1 week before the experimental processes. All experimental procedures were performed in compliance with the National Institutes of Health guidelines and were approved by the Animal Care and Use Committee of the Peking University Health Science Center (LA2021029).

### Cell Culture

Human normal pancreatic cells and pancreatic TCs (AsPC-1, PANC1 and SW1990) were obtained from the National Infrastructure of Cell Line Resource (NICR, Beijing, China) and were cultured in Roswell Park Memorial Institute Medium (RPMI) 1640 medium (Invitrogen, MA, United States) containing 10% fetal bovine serum (FBS, Gibco, MA, United States). Human umbilical vein endothelial cells (HUVECs) were procured from the American Type Culture Collection (ATCC, VA, United States) and were cultured in endothelial cell medium (ECM) medium (Sciencell, CA, United States) containing 20% FBS.

### Analyses of Lung Metastasis in Animal Models

Hematogenous metastasis of PC cells was simulated by intravenously injecting human pancreatic TCs AsPC-1 (1 × 10^6^ cells/mouse) into BALB/C-nu/nu nude mice through the tail vein as previously described (Tiwari et al., 2020). To determine the effect of BBR on the survival of the model mice, the mice were randomly divided into three groups, namely, the control group, lower concentration group (100 mg/kg/day), and higher concentration group (200 mg/kg/day). Oral gavage with vehicle or BBR was performed 3 days before intravenous injection of AsPC-1 cells. The survival status of the mice was monitored every day, while their body weights were recorded every 2 days. To evaluate the effects of BBR on PC metastasis, the mice were randomly assigned into two groups. The first group was subjected to oral gavage with vehicle, while the second received BBR (200 mg/kg/day) treatment *via* oral gavage. BBR treatment was initiated 3 days before the injection of mice with AsPC-1 cells. Six weeks later, the mice were sacrificed *via* overdose by intraperitoneally injecting pentobarbitone sodium (100 mg/kg). Mouse plasma, lungs, livers, and kidneys were harvested. Lung metastasis was evaluated by examining lung paraffin sections stained with H&E. Briefly, whole lungs were embedded with paraffin, and coronal sections were harvested. Sections with a maximum lung area were chosen for lung metastasis comparisons. Tumor foci of different groups were counted randomly by a pathological expert. Areas of tumor foci were determined using the Image J software (v1.52p, National Institutes of Health, MD, United States).

### Flow Cytometric Analysis of Lung Infiltration of Circulating Tumor Cells

Mice were randomly divided into two groups: the control group and BBR (200 mg/kg/day) group. The mice in the two groups were respectively gavaged with a vehicle of BBR for 3 days. Carboxyfluorescein succinimidyl ester (CFSE)-labeled single tumor cell suspensions (AsPC-1, 1 × 10^6^ cells/mouse) were intravenously injected into the mice through the tail vein. The mice were then sacrificed *via* overdose with pentobarbitone sodium (100 mg/kg) 24 h after injection. The whole lung was perfused with cold phosphate buffered saline (PBS) through the right ventricle using a syringe until the pulmonary vasculature was cleared of blood to remove the remaining circulating CFSE-labeled AsPC-1 cells. The right lung was then resected immediately and lysated to create a pulmonary single cell suspension as previously described (Hao et al., 2017). The percentages of CFSE-labeled TCs were measured using the Cytoflex flow cytometer (Beckman, CA, United States).

### Confocal Fluorescence Imaging of the Lung Tissue Section

Mice were randomly divided into two groups: the control and BBR (200 mg/kg/day) groups, and the following procedure including gavage, tail vein injection of CFSE-labeled AsPC-1 cells, euthanasia and clear of pulmonary vasculature are the same with that for flow cytometry analysis. Lungs were collected and fixed in 4% paraformaldehyde at room temperature for at least 24 h. The tissues were subsequently sectioned into 20 μm frozen slices using a microtome and transferred into adhesive slides. After blocking with 5% normal goat serum (Solarbio), slices were stained using CD31 antibody (rabbit mAb, NB100-2284, Novus Biologicals) and Alexa Fluor at 568 tagged secondary antibodies (goat anti-rabbit, ab 175471, Abcam). Fluorescence imaging and Z-stack imaging for the 3D reconstruction of a region of interest were performed on a confocal LSM microscope (Zeiss, LSM 880).

### Trans-Endothelial Migration Assay

The Transwell system (Corning, NY, United States) was used to analyze the trans-endothelial migration of TCs. Briefly, HUVECs were planted and maintained on the upper chamber (pore size, 8 μm; 8,000–10,000 cells/well) for 20 h. After their stabilization, the endothelial layer was stimulated with unlabeled PC cells using a vehicle, BBR, or plus SD-208 (10 μM) for another 20 h. The trans-endothelial migration of TCs was determined by replacing the medium in the upper chamber with FBS-free 1640 medium containing CFSE-labeled AsPC-1 cells (10,000 cells/well) and by replacing the medium in the lower chamber with 1640 medium containing 20% FBS. After 24 h, the cells in the bottom of the upper chamber were fixed with 4% paraformaldehyde for 10 min, while the cells in the inner upper chamber were scratched. The CFSE-AsPC-1 cells that underwent trans-endothelial migration were then visualized and photographed using an inflorescent microscopy (Leica, Weztlar, Germany). For each well, five random photos were taken. The CFSE-positive cells in the resulting images were counted using the Image-Pro Plus software, version 6.0 (Media Cybernetics, Inc. Rockville, MD, United States).

### Surface Plasmon Resonance Analysis

The recombinant TGFBR1 (Sino Biological, Beijing, China) was used for SPR analysis using a Biacore T200 instrument (Biacore, Uppsala, Sweden). PBS-P (20 mM PBS, pH 7.4, 2.7 mM KCl, 137 mM NaCl, 0.05% Surfactant P20) was utilized as a running buffer at a flow of 10 μl/min at 25°C. The CM5 sensor chip was activated using 1:1 NHS/EDC for 7 min. The His-tagged TGFBR1 (Sino Biological, Beijing, China) was immobilized on the CM5 chip at a concentration of 10 μg/ml in 10 mM sodium acetate with a pH of 5.5. Amine-coupling occurred until a surface density of 10,000 RU was achieved. Binding analyses were carried out at 25°C with a flow rate of 30 μl/min. BBR in a running buffer (1 × PBS, 0.05% Tween 20, and 5% dimethyl sulfoxide; pH 7.4) was added to TGFBR1 at gradient concentrations of 1.5625, 3.125, 6.25, 12.5, 25, and 50 μM. An empty flow cell without any immobilized protein was used as a deducted reference. The binding curves were analyzed using a kinetic binding model in the Biacore Evaluation Software (GE Healthcare, MA, United States).

### Transforming Growth Factor-β Receptor 1 Kinase Activity Assay

To characterize the TGFBR1 kinase potency of BBR, the IC_50_ of TGFBR1 was determined by Invitrogen (Life Technologies, MA, United States) using the fluorescence resonance energy transfer (FRET)-based Z-LYTE SelectScreen Kinase Profiling Service.

### Molecular Simulation

The molecular docking between BBR and TGFBR1 (PDB ID: 3HMM) was simulated using an artificial intelligence drug target screening system (http://www.dtspace.tech), which was based on the Discovery Studio 2021 (DS; BIOVIA-Dassault Systèmes). The selected poses of BBR with TGFBR1 were subjected to 100 ns molecular dynamics (MD) simulations using DS. The stability of the complex was analyzed and confirmed by plotting the root mean square deviation (RMSD). The RMSD was a measure of the deviation of the conformational stability of the proteins from their backbone structures to their early starting structures and an estimate of the fundamental property investigation in MD. The binding energy of the stable complex was calculated using the Chemistry at Harvard Macromolecular Mechanics (CHARMM) force field, which represented the sum of electrostatic and van de Waals interaction terms.

### Western Blotting

Western blotting was conducted to determine the effect of BBR on TGF-β1 signaling in ECs. HUVECs were cultured in 6-well plates and treated with TGF-β1 (10 ng/ml) or TGF-β1 with 3, 10, or 30 μM of BBR for 24 h in RPMI 1640 medium containing 1% FBS. Cells were then lysed with radioimmunoprecipitation assay buffer (RIPA) buffer (Invitrogen, Carlsbad, CA, United States) after being washed with PBS thrice. Immunoblots were then quantified as described previously (Hao et al., 2017). The primary antibodies used in this study were as follows: rabbit anti-phospho-Smad2 antibody (1:1000, CST, MA, United States), rabbit anti-phospho-Smad3 antibody (1:1000, CST), and mouse anti-actin antibody (1: 10,000, TDYbio, Beijing, China).

### Quantitative Real-Time Polymerase Chain Reaction

RT-PCR was conducted to quantify the expression levels of TGF-β1 in various types of PC cells and to determine the effect of BBR on TGF-β1 signaling in HUVECs that were subjected to the same procedures as those in western blotting analysis. mRNA was extracted using TriZol (Thermo Scientific, MA, United States) following the manufacturer’s instructions. Quantitative gene expression analysis was performed using SYBR Green Master Mix on a 7500 Fast Real-Time PCR system (Applied Biosystems, MA, United States). The primers used are presented in Table 1.

**TABLE 1:** Primers used in this study.

Gene	Primer Sequence (5′–3′)
*TGF-β1*	F: CCG​ACT​ACT​ACG​CCA​AGG​AGG​T
	R: TCA​ACC​ACT​GCC​GCA​CAA​CTC
*SNAI1*	F: TTC​GCT​GAC​CGC​TCC​AAC​CT
	R: CCA​GGC​AGA​GGA​CAC​AGA​ACC​A
*Slugik*	F: AGC​ACA​CTG​AGT​GAC​GCA​ATC​A
	R: TTG​GTT​GGT​CAG​CAC​AGG​AGA​A
*GAPDH*	F: TGA​CCA​CAG​TCC​ATG​CCA​TCA​CT
	R: ACG​CCT​GCT​TCA​CCA​CCT​TCT

### Enzyme-Linked Immunosorbent Assay

The plasma levels of TGF-β1 were determined *via* ELISA using a commercial ELISA kit (DB100B, R&D Systems, Inc., MN, United States), which was suitable for examining both human and mouse TGF-β1. The manufacturer’s instructions were followed. Absorbance was measured at a wavelength of 450 nm using an EnSpire Multilable Reader (2300, PerkinElmer, MA, United States).

### Immunofluorescence Staining

For immunofluorescence, the paraffin-embedded tissues were cut into 5 μm sections to be deparaffinized in xylene, and rehydrated with ethanol; heat-induced antigen retrieval was achieved by boiling in EDTA antigen retrieval water (PH 9.0; ZSGB-BIO, Beijing, China). The slides were then incubated with a goat serum with 0.25% Triton solution for blocking. Following incubation with monoclonal anti-CD31 (1:200; Abcam), monoclonal anti-Smad2 (phospho S255; 1:200; Abcam), monoclonal anti-Smad3 (phospho S423 + S425; 1:200; Abcam), and polyclonal anti-TGF beta receptor I (1:200; Abcam) primary antibodies overnight at 4°C, these slides were incubated with Alexa Fluor-594-coupled and/or Alexa Fluor-488-coupled secondary antibodies for 3 h at room temperature. The VectaShield antifade media with DAPI was used to stain nuclei. These slides were imaged under a Zeiss inverted fluorescence microscope (AXI0; Zeiss) equipped with a Zen software or using a laser-scanning confocal microscope (SP8; Leica) with a ×20 water immersion objective. All images were statistically analyzed with the Image-Pro Plus 6.0 software (Media Cybernetics, Inc. Rockville, MD, United States).

### Statistical Analyses

GraphPad Prism eight software (version 8.0.2, GraphPad Software, Inc., San Diego, CA, United States) was used for statistical analyses. The Student’s *t*-test was conducted to determine the differences between two groups. One-way analysis of variance (ANOVA) with a post-hoc test was used to compare three or more groups. Two-way ANOVA was performed to analyze the body weight changes of the mice during BBR treatment. Results were expressed as means ± SEM. Differences were considered statistically significant at *p* < 0.05.

## Data Availability

The datasets presented in this study can be found in online repositories. The names of the repository/repositories and accession number(s) can be found below: https://www.jianguoyun.com/p/DXh8xosQx6u7Chj72bgEIAA.
