# Supplementary material for: Berberine Suppresses Lung Metastasis of Cancer via Inhibiting Endothelial Transforming Growth Factor Beta Receptor 1
Source: Front Pharmacol. 2022 Jun 16;13:917827. doi: 10.3389/fphar.2022.917827 (PMC9243563; doi:10.3389/fphar.2022.917827)
Supplement: Supplementary file 1 [file DataSheet1.docx]

***Supplementary figures and figure legends***

**Berberine suppresses tumor cells lung metastasis by inhibiting endothelial transforming growth factor beta receptor 1**

Wenjia Tian^#^, Huifeng Hao^#^, Ming Chu,^#^, Jingjing Gong, Wenzhe Li, Yuan Fang, Jindong Zhang, Cunzheng Zhang, Yonghui Huang, Fei Pei, Liping Duan^∗^


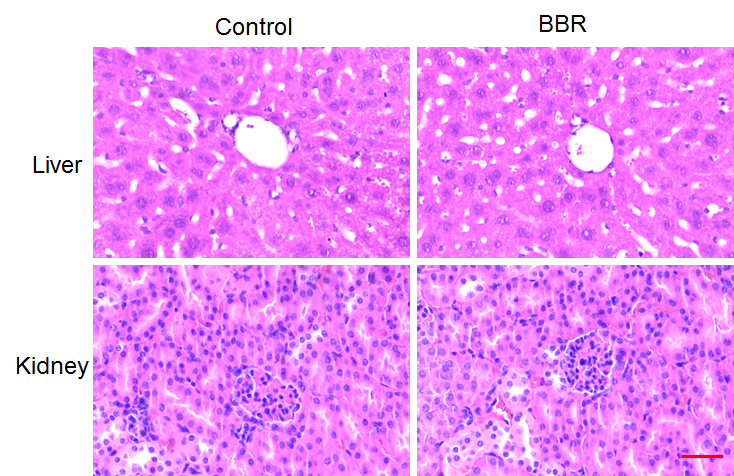


**Supplementary Fig. 1: Effects of BBR on the morphology of mice livers and kidneys.** Representative images of hematoxylin-eosin staining of livers and kidneys of mice treated with the vehicle or BBR (200 mg/kg). Scale bar = 50 μm.


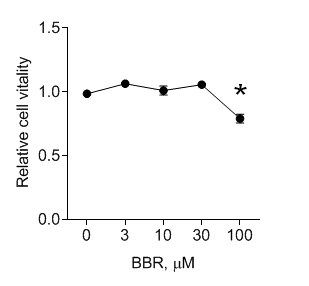


**Supplementary Fig. 2: Effects of BBR on proliferations of SW1990 cells.** SW1990 cells were treated with BBR at indicated concentrations for 24 hours. One way ANOVA with Sidak's multiple comparisons test; n = 4.


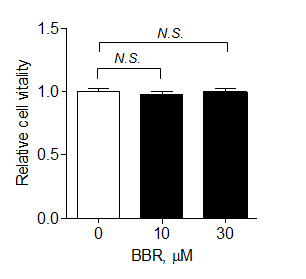


**Supplementary Fig. 3: Effects of BBR on endothelial cell viability *in vitro*.** n = 6, one-way ANOVA.


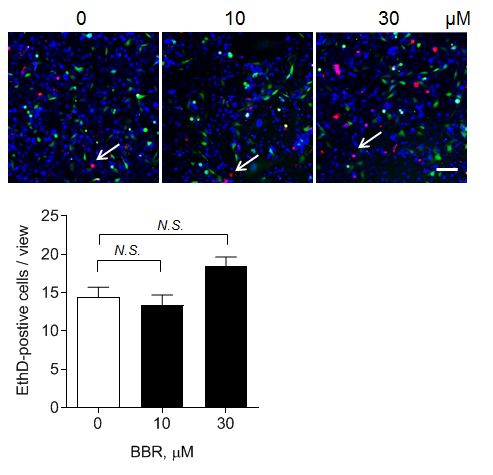


**Supplementary Fig. 4: *In vitro* effect of BBR on EC necroptosis.** Green represents CFSE-labeled PC cells, red represents EthD-Ⅲ-positive cells, blue represents the DAPI-stained nuclei, white arrows denote the representative ECs that are undergoing necroptosis. n = 15, one-way ANOVA. Scale bar = 100 μm

**
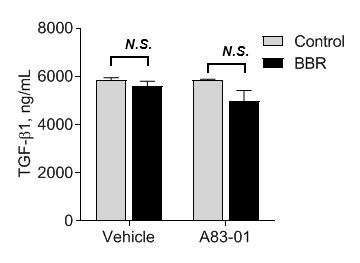
**

**Supplementary Fig. 5: Serum TGF-β1 levels in the vehicle- or A83-01-treated groups.** n = 6 in vehicle group, n = 4 in the A83-01 group, one-way ANOVA.


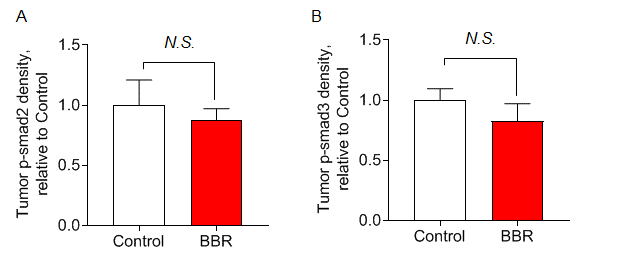


**Supplementary Fig. 6: Effects of BBR on phosphorylations of smad2/3 in cancer cells *in vivo*.** Statistical results of the effects of BBR on phosphorylations of SMAD 2 (A) and SMAD 3 (B) in cancer cells. Student’s t-test; n = 5.


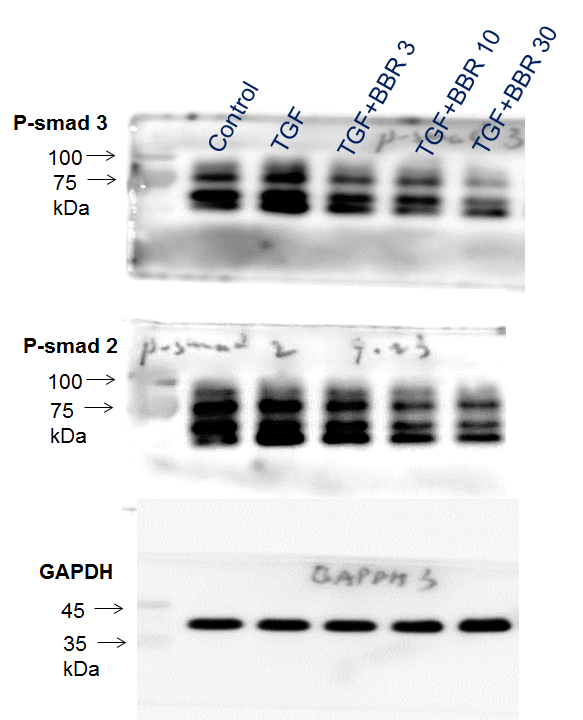


**Supplementary Fig. 7: Uncropped Western blot pictures for Figure 4D.**


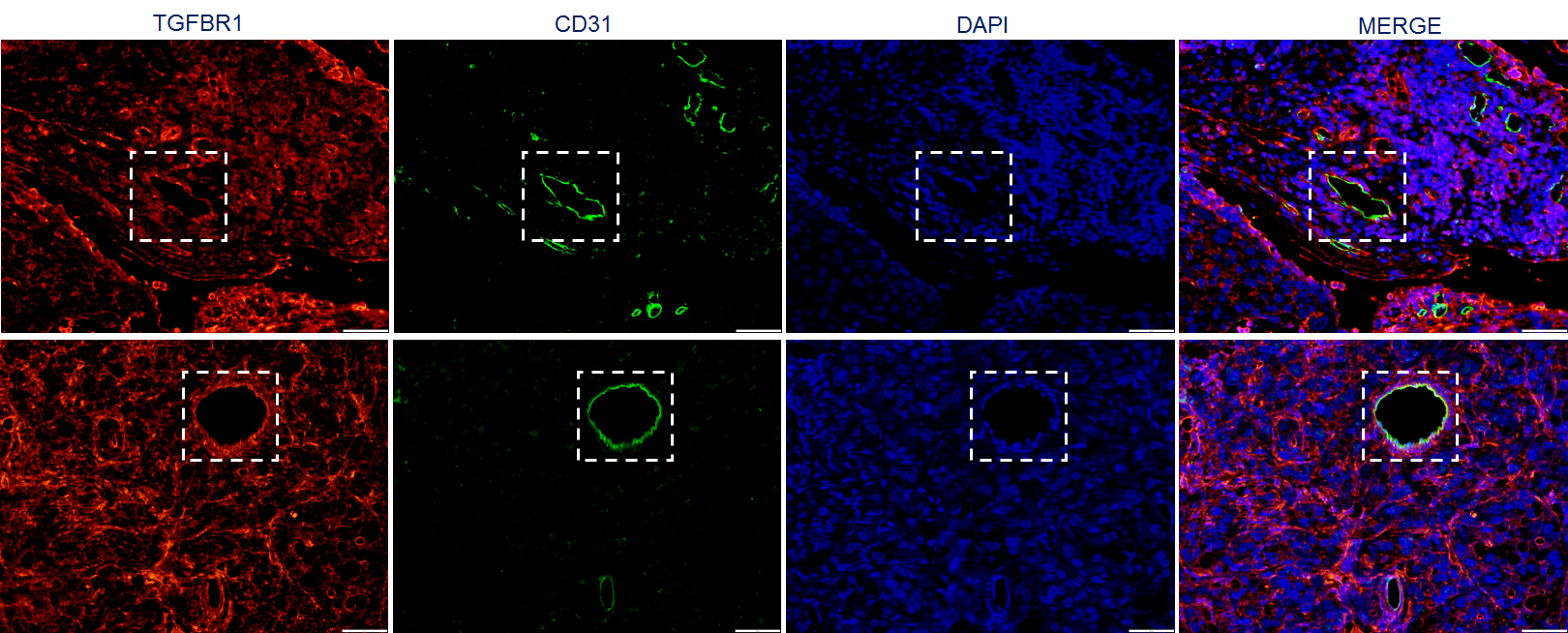


**Supplementary Fig. 8: Uncropped Western blot pictures for Figure 6D.** To clearly exhibit TGFBR1 signals in the endothelial cells (CD31 positive cells), images inside the dotted line were shown in the Figure 6D in the paper.


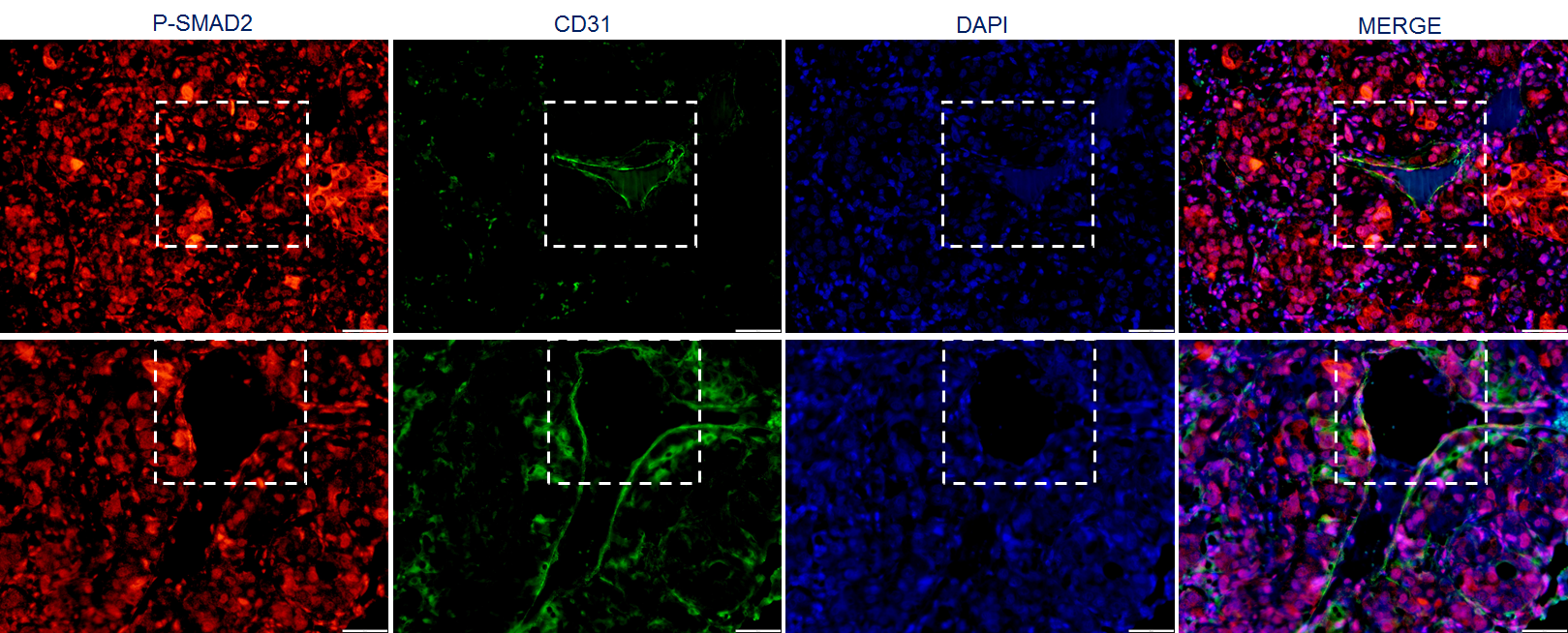


**Supplementary Fig. 9: Uncropped Western blot pictures for Figure 6E.** To clearly exhibit P-SMAD2 signals in the endothelial cells (CD31 positive cells), images inside the dotted line were shown in the Figure 6E in the paper.


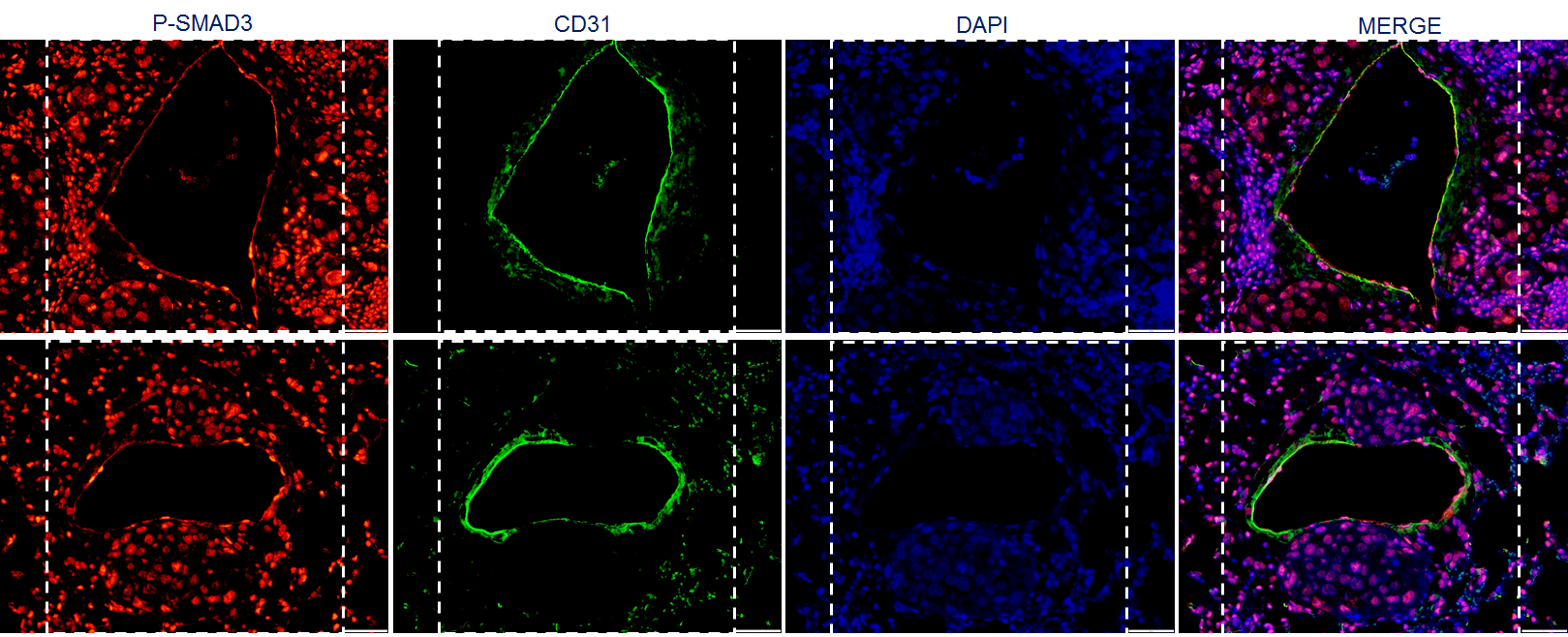


**Supplementary Fig. 10: Uncropped Western blot pictures for Figure 6F.** To clearly exhibit P-SMAD3 signals in the endothelial cells (CD31 positive cells), images inside the dotted line were shown in Figure 6F in the paper.
